# Social Support and Hope Mediate the Relationship Between Gratitude and Depression Among Front-Line Medical Staff During the Pandemic of COVID-19

**DOI:** 10.3389/fpsyg.2021.623873

**Published:** 2021-03-10

**Authors:** Lijuan Feng, Rong Yin

**Affiliations:** ^1^Student Mental Health Education and Counseling Center, Northwest Minzu University, Lanzhou, China; ^2^Department of Neurology, The 940th Hospital of Joint Logistics Support Force of the PLA, Lanzhou, China

**Keywords:** COVID-19, gratitude, social support, hope, depression

## Abstract

**Background:**

The pandemic of Coronavirus Disease 2019 (COVID-19) has burdened an unprecedented psychological stress on the front-line medical staff, who are at high risk of depression. While existing studies and theories suggest that factors such as gratitude, social support, and hope play a role in the risk of depression, few studies have combined these factors to explore the relationship between them.

**Objective:**

This study examined the mediating roles of social support and hope in the relationship between gratitude and depression among front-line medical staff during the pandemic of COVID-19.

**Methods:**

This study used the Gratitude Questionnaire, the Perceived Social Support Scale (PSSS), the State Hope Scale (SHS), and the Center for Epidemiologic Studies Depression Scale to examine the gratitude, social support, hope, and depression among 344 front-line medical workers in Wuhan, which was the hardest-hit area of COVID-19 in China.

**Results:**

The results showed that the prevalence of mild depressive disorder was 40.12% and the prevalence of major depressive disorder was 9.59% among front-line medical staff during the pandemic of COVID-19; gratitude has a direct and negative effect on depression. Gratitude was negative predictors of depression through the mediating variables of social support and hope [β_*gratitude*__–__*social support*__–__*depression*_ = −0.096, 95%CI(−0.129 to −0.064); β_*gratitude*__–__*hope*__–__*depression*_ = −0.034, 95%CI(−0.055 to −0.013)], as well as via an indirect path from social support to hope [β_*gratitude*__–__*social support*__–__*hope*__–__*depression*_ = −0.089, 95%CI (−0.108 to −0.070)].

**Conclusion:**

The study findings indicate that gratitude as a positive emotion can reduce depression in medical staff by promoting social support and hope, respectively. Gratitude also reduced depression in health care workers through a chain mediating effect of social support and hope. Overall, gratitude can directly foster social support and hope, and protect people from stress and depression, which has implications for clinical interventions among front-line medical staff during the pandemic of COVID-19.

## Introduction

Previous studies have found that in high-risk and stressful pandemic environments, medical staff are prone to have a range of psychological problems (Bohlken et al., 2020). Coronavirus Disease 2019 (COVID-19) is highly contagious and has a certain mortality rate, and individual’s anxiety that he or she might be infected with novel coronavirus may result in the appearance of negative emotions, which can lead to depressive symptoms and other psychological problems (Spoorthy, 2020). The medical staff in the frontline of pandemic have to deal with the social and psychological stress of high exposure to the virus while working to treat patients. Therefore, the mental health of medical staff has become a worldwide topic that is worthy of attention, and how to reduce the depression symptoms of medical staff has become one of the key challenges to deal with the pandemic. According to the previous studies, some positive emotions are the protective factors of depression in stressful situations, among which gratitude, as a common positive emotion, may play an important role in reducing depression (Fredrickson, 2001).

Within the field of gratitude research, there is a lack of agreement about the nature of the concept. Gratitude has been conceptualized as an emotion, an attitude, a moral virtue, a habit, a personality trait, or a coping response (Emmons and Shelton, 2002; Emmons and McCullough, 2003). In part, gratitude is an emotion that occurs after people receiving aid which is perceived as costly, valuable, and altruistic (Wood et al., 2008a). On this basis, current studies have conceptualized gratitude as an emotion that is always directing toward appreciating the helpful actions from other people (McCullough et al., 2002). Wood et al. (2008a) showed that feeling grateful for the positive aspects of the world would be likely to make a depressive bout more bearable and of shorter duration. Seligman et al. (2005) suggested that by increasing gratitude, depression could be effectively reduced.

The broaden-and-build theory of positive emotions argues that gratitude may build up social resources and psychological resources (Fredrickson, 2001). These resources function as reserves to be drawn in times of need, which is beneficial during the difficult periods of people lives (Emmons and McCullough, 2003). On one hand, the building of social resources can improve the interaction mode between the individual and the external environment, help individuals to establish a more supportive social system, and make individuals feel and accept the support of others (Deichert et al., 2019). On the other hand, the building of psychological resources can help individuals to face the future with a more positive attitude and have more hope for the future (Loo et al., 2014).

The experience of gratitude and the actions stimulated by it build and strengthen social bonds and friendships. Moreover, encouraging people to focus on the benefits they have received from others leads them to feel loved and cared for by others (McCullough et al., 2008; Lan and Wang, 2019a). Therefore, gratitude appears to build friendships and other social bonds. These are social resources because, in times of need, these social bonds are wellsprings to be tapped for the provision of social support (Emmons and McCullough, 2003). Adequate social support can provide a safe environment for individuals to talk freely with others about negative experiences and related emotions, thus reducing individual depression symptoms (Hobfoll and Shirom, 2001; Lan and Wang, 2019b). It is also found that social support can reduce depression according to relevant empirical studies (Lan et al., 2019).

In addition to building up social resources, gratitude can also reduce depression by building up psychological resources, such as hope (Fredrickson, 2001). Gratitude is a positive evaluation of the benefits that already obtained, while hope is a positive expectation of the expected results in the future (Scioli et al., 2011). McCullough et al. (2002) argued that people who are full of gratitude and hope will enjoy their lives, and whether they look at the past positively or pursue meaningful future goals, the inner social orientation of gratitude may further promote the generation of hope. Witvliet et al. (2019) found that by letting the individuals recall people or things that deserve gratitude in the past, their hope for the future could be effectively improved. Increasing individuals’ hope can help them to distract their attention from negative events, and promote individuals to adopt more adaptive strategies to deal with negative events, thus alleviating depression symptoms (Snyder, 2002; Kaleta and Justyna, 2020). An empirical study on post-traumatic groups also found that the higher the level of hope, the lower the level of depression (Hassija et al., 2012).

Social support not only provides material support to make up for the resources that individuals lose in coping with stress, but also improves their sense of meaning and sense of purpose (Wang et al., 2020). According to the theory of social connectedness, social connectivity represented by “keeping close relationship with society” can meet the individuals’ belonging needs, and can provide support for the individuals’ goal-oriented behavior, thus promoting the generation of hope (Lee et al., 2001).

### The Present Study

Since the COVID-19 outbreak, the growing number of patients has put tremendous pressure on the local medical system and medical staff in Wuhan. Medical workers in Wuhan have been facing many challenges (Kang et al., 2020). At the time when local medical supplies and staff were in short supply, medical workers from other provinces of China rushed to Wuhan for assistance after January 23, 2020. As of April, a total of 42,000 medical staff from all over China had arrived Wuhan. Gansu Province sent a medical team of several hundred people to Wuhan to participate in the treatment of COVID-19 patients. This study investigated the incidence of depression among front-line medical staff participating in the treatment of COVID-19 patients from Gansu Province and the influence mechanism of gratitude on depression.

In recent years, with the rise of positive psychology, more and more researchers have begun to pay attention to the positive influence that negative events may bring to individuals. Seligman and Csikszentmihalyi (2000) stated that the study of positive psychology should focus on positive emotion, positive environment and positive attitude. Gratitude as a positive emotion may influence depression through a combination of positive environmental factors represented by social support and positive attitude represented by hope (Wang and Wu, 2020). Although previous studies have explored the relationship between gratitude, social support, hope, and depression, respectively, few studies have examined how gratitude affects depression through the mediating role of social support and hope. From the perspective of positive psychology, this study intends to investigate the influence of gratitude on depression of medical staff during the pandemic, and analyze the mediating effect of social support and hope. On the basis of the previous theoretical and empirical studies, this study assumes that gratitude can directly and negatively predict depression, and can also positively predict depression through the intermediary of social support and hope, and can also positively predict depression through the chain intermediary of social support and hope.

## Methods

### Participants and Procedures

Participants in this study involved 344 front-line medical workers in Wuhan, which was the hardest-hit area of COVID-19 in China. The medical workers were from a medical team from Gansu province that aimed to assist Wuhan city. The research team recruited them online and offline after obtaining the consent of the leader of the assistance medical team. These medical workers had been on the frontline of treating COVID-19 for more than 2 months prior to participating in the present study. The period of data collection lasted 10 days and was undertaken between April 27, 2020 and May 6, 2020. A total of 360 medical workers participated in our survey, and 344 (95.56%) of them completed all the questionnaires.

The Institutional Ethics Committee approved all the procedures. The purpose of the study and the autonomy of medical workers were highlighted before the survey. Written informed consent forms were obtained from each participant, and the participants were free to withdraw from the survey at any time. Once recruited and consented, the participants then completed the survey through the Wenjuanxing platform which is an online survey tool.

### Measures

#### Gratitude

Gratitude was measured using Gratitude Questionnaire-6 (GQ-6, McCullough et al., 2002). This questionnaire includes six items, each of the items are scored on a seven-point scale ranging from 0 (completely disagree) to 6 (completely agree), for example, “I have so much in life to be thankful of.” Higher scores represent higher levels of gratitude. This scale shows good reliability in Chinese (Wang et al., 2018). Cronbach’s alpha was 0.86 in the current study.

#### Social Support

Perceived social support was measured by the Perceived Social Support Scale (PSSS; Zimet et al., 1988), which was validated in the Chinese context before by Chou (2000), showing adequate concurrent and construct validity. The PSSS is a 12-item self-report scale that assessing perceived support arising from three dimensions, namely, family support (e.g., “I get the emotional help and support I need from my family”), friend support (e.g., “I can count on my friends when things go wrong”), and others support (e.g., “There is a special person in my life who cares about my feelings”). Each item is scored on a seven-point scale ranging from 1 (completely disagree) to 7 (completely agree). Total scores can range from 12 to 84, with higher scores indicating greater perceived social support. In the present study, the Cronbach’s alpha was 0.87.

#### Hope

Hope was assessed using the State Hope Scale (SHS; Snyder et al., 1996). This scale includes six items that assess agency (e.g., “I meet the goals that I set for myself”) and pathways thinking (e.g., “I can think of many ways to get out of a jam”), ranged from 1 (completely disagree) to 8 (completely agree). A higher score indicates a higher degree of sense of hope. The Chinese version of the SHS has been proven to be a valid scale (Zhou et al., 2017). In the present study, the Cronbach’s alpha was 0.83.

#### Depressive Symptoms

Depressive symptoms were measured by the Center for Epidemiological Studies-Depression Scale (CES-D, Radloff, 1977). This scale has 20 items (e.g., “I was bothered by things that don’t usually bother me”), and the Chinese version has been well validated (Lan and Wang, 2019b). The items are rated on a four-point scale that ranges from 0 (rarely) to 3 (sometimes). After four items are reversed coded, a higher total score indicates a higher level of depressive symptoms. Cronbach’s alpha was 0.86 in the current sample.

### Data Analysis Strategies

Data analyses were conducted via SPSS 17.0 and Mplus 7.0. First, descriptive analyses were conducted for the variables of interest for the total sample. Structural Equation Model (SEM) was carried out to examine the mediating role of social support and hope in the relationship between gratitude and depression among front-line medical staff during the pandemic of COVID-19. Missing data were handled with full-information maximum likelihood (FIML) estimates in structural models. We used chi-square values to evaluate model fit, the comparative fit index (CFI), the Tucker–Lewis index (TLI), the RMSEA, and the standardized root-mean residual (SRMR). A non-significant chi-square indicates good model-data fit. The general cutoffs for accepting a model are equal to or greater than 0.90 for the CFI and TLI, and less than 0.08 for the SRMR and RMSEA (Wen et al., 2004).

**FIGURE 1:**
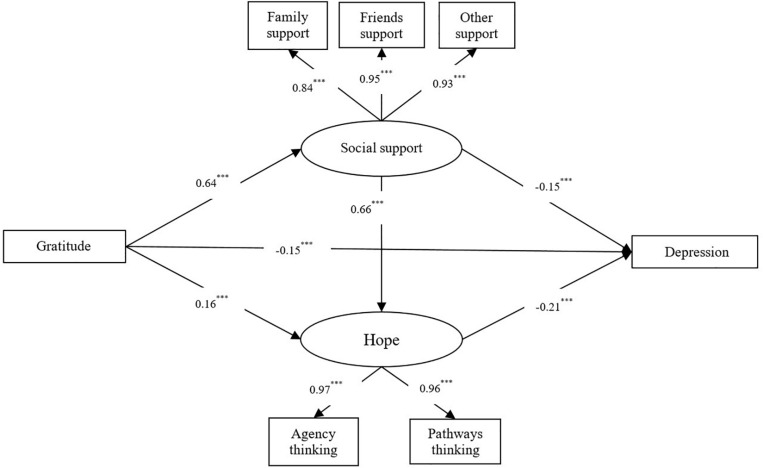
The multiple indirect effects model. ^∗∗∗^*p* < 0.001.

Measurement model fit should be evaluated before proceeding to an evaluation of the full model (Anderson and Gerbing, 1988). This stepwise procedure offers the safeguard of explicitly verifying the acceptability of construct measurement before proceeding to an evaluation of relationships among constructs. We followed this approach here. Next, we applied the SEM approach to assess the following models: (a) a direct effect model with structural paths from gratitude to depression and (b) an indirect effect model, with the mediators (social support and hope) inserted between gratitude and depression, and one predictive path from social support to hope added. The final model is presented in Figure 1.

## Results

### Characteristics of Participants

Sample characteristics data from 344 front-line medical workers were included in the analysis. The mean age was 34.05 years (SD = 7.25, range 20–57), 65.4% of the participants were females, 34.6% were males, average age were 34.05 years (*SD* = 7.25; min 20 years old—max 57 years old). Of the 344 participants included in this study, 40.1% were medical doctors and 59.9% were nurses.

### Descriptive Statistics and Correlations

As shown in Table 1, gratitude was significantly and positively associated with social support and hope, negatively associated with depression; social support was significantly and positively associated with hope, negatively associated with depression; hope was significantly and negatively associated with depression. In addition, the results showed that the prevalence of mild depressive disorder was 40.12% and the prevalence of major depressive disorder was 9.59% among front-line medical staff during the pandemic of COVID-19.

**TABLE 1:** Descriptive statistics and correlations for key variables (*N* = 344).

Variables	*M*	*SD*	Gratitude	Social support	Hope	Depression
**Gratitude**	27.19	6.43	1			
**Social support**	63.34	15.07	0.63***	1		
**Hope**	35.55	8.78	0.56***	0.73***	1	
**Depression**	14.87	8.97	−0.31***	−0.31***	−0.36***	1

***p < 0.001.

### Structural Equation Model Analyses

First, the measurement model was tested. It consisted of two latent variables: social support and hope. The social support latent variable has three subscales: family support, friends support, and other support. The hope latent variable has two subscales: agency thinking and pathways thinking. The measurement model fit was acceptable: χ^2^/df = 2.885, CFI = 0.951, TLI = 0.976, RMSEA (90% CI) = 0.035(0.011–0.059).

Prior to testing the mediating effect, we examined the direct effect of gratitude on depression. The direct effects model demonstrated a good fit: [χ^2^/df = 2.521, CFI = 0.948, TLI = 0.972, RMSEA (90%CI) = 0.052 (0.033–0.071)]. The results of the path analysis indicated that the path coefficient of gratitude direct effect on depression was β = 0.372 (*p* < 0.001).

In order to further probe into the predictive mechanism of gratitude on depression, in this study, social support and hope were simultaneously included as mediating variables in the direct effect model based on a direct model. The fit index of the model was considered ideal [χ^2^/df = 3.985, CFI = 0.927, TLI = 0.951, RMSEA (90% CI) = 0.067 (0.060–0.075)]. The results of the model are shown in Figure 1. Analysis on the various paths in the model found that gratitude was negative predictors of depression through the mediating variables of social support and hope [β_*gratitude*__–__*social support*__–__*depression*_ = −0.096, 95%CI (−0.129 to −0.064); β_*gratitude*__–__*hope*__–__*depression*_ = −0.034, 95%CI(−0.055 to −0.013)], as well as via an indirect path from social support to hope [β_*gratitude*__–__*social support*__–__*hope*__–__*depression*_ = −0.089, 95%CI (−0.108 to −0.070)].

## Discussion

This study found that the depression was common among front-line medical staffs during the pandemic of COVID-19. Medical staffs have to identify the people infected with the disease, respond to their treatment needs, carry out the severe and difficult treatment processes in hospitalized patients, face the psychological breakdown caused by each patient passed away, and also face the risk of developing the disease at any time (Seçer et al., 2020). These factors will lead to an increased risk of depression among medical staff.

However, this study found that gratitude can reduce the depression of medical staff, which is consistent with previous studies (McCullough et al., 2002; Lin, 2015). From the perspective of broaden-and-build theory of positive emotions, gratitude, as a typical positive emotion, helps individuals to look at others and the world from a more positive perspective, thus reducing individual depression (Fredrickson, 2001). Through further research on the mechanism of gratitude affecting depression, it is found that social support and hope play an intermediary role in the influence of gratitude on depression.

This study found that gratitude can reduce depression by promoting social support. The promotion of gratitude to social support is consistent with previous studies (Wang et al., 2018). Gratitude may lead to the development of more supportive environments, represented in conscious awareness as perceived social support (Wood et al., 2008b). Additionally, gratitude leads to characteristic attributions regarding social situations, with grateful people interpreting the help they receive as more valuable, more costly, and seeing their benefactors’ intentions as more altruistic (Wood et al., 2008a; Algoe et al., 2013). As gratitude is involved in both encouraging actual supportive behaviors and in appraising situations positively, gratitude seems particularly likely to lead to perceived social support (Wood et al., 2008a; Kong et al., 2015).

By analyzing the influence of gratitude on depression path through hope, we can find that gratitude may lower the occurrence of depression by improving the hope of medical staff. From the relationship between gratitude and hope, gratitude is a positive evaluation of what has happened, while hope is a positive expectation of what has not happened (Scioli et al., 2011). People who are grateful in their lives mean that they have a more positive evaluation of their past, and these people are often more able to look at the future positively and have a higher sense of hope (McCullough et al., 2002). Previous research has also found that being grateful to people who helped them in the past can help individuals cope with future difficulties and challenges in a more positive attitude and make them hopeful about the future (Witvliet et al., 2019). During the pandemic, the hope level of medical staff is particularly important for depression. The pandemic may lead to the breakdown of the medical staff’s original cognitive beliefs about the world, lead to the loss of the medical staff’s sense of control over themselves, others or the world, and even lead to the loss of hope for the future (Glass et al., 2009). The hope aroused by gratitude can make the medical staff who affected by the pandemic distract their attention from the clues such as negative news reports related to the pandemic, and urge individuals to pay attention to more positive information. In addition, hope can make the medical staff cope with a series of frustration experiences better during the pandemic, help them discover the positive connotation behind the disaster, and promote them to fully explore their own potential to deal with difficulties, and finally play a role in reducing depression (Snyder, 2002; Hassija et al., 2012; Kaleta and Justyna, 2020).

This study also found that gratitude can reduce depression through the chain mediation of social support and hope. During the pandemic period, the sense of belonging of medical staff with more social support will be better satisfied, and their goal-oriented behavior will be fully stimulated, which will help to enhance the hope of medical staff for future life (Lee et al., 2001).

### Implications for the Clinical Practice

This study suggests that psychological intervention workers should not only pay attention to alleviating the negative psychological problems of medical staff, but also pay attention to the positive psychological growth of medical staff. Practices such as gratitude writing and gratitude visit can be used to promote gratitude and to guide medical staff to the people and things for which they are grateful (Seligman et al., 2005). It can also guide medical staff to further transform gratitude and sufficient social support into psychological resources facing the future, so that medical staff can actively cope with the difficulties during the pandemic through the hope for the future.

### Limitations

However, there are some limitations in this study. First of all, this study focuses on the medical staff aiding Wuhan who are affected by the pandemic situation, and these medical staff have different degrees of trauma exposure in the pandemic situation. The study does not adequately control for other factors that may influence depression, such as whether the medical staff was in a satisfying relationship. Second, cross-sectional data are used in this study, which makes it difficult to explain the causal relationship between variables. Previous studies, for example, have suggested that closer social connections may help people develop gratitude (Alfieri et al., 2018). Future studies should investigate further from the perspective of tracking. Finally, according to this study, it is found that after adding social support and hope, the direct prediction effect of gratitude on depression in the model is still significant, which indicates that there may be other factors that play a role in the influence of gratitude on depression, and future research can further examine the mechanism from other perspectives.

## Conclusion

The study findings indicate that gratitude as a positive emotion can reduce depression in medical staff by promoting social support and hope, respectively. Gratitude also reduces depression in health care workers through a chain mediating effect of social support and hope. Overall, gratitude can directly foster social support and hope, and to protect people from stress and depression, which has implications for clinical interventions among front-line medical staff during the pandemic of COVID-19.

## Data Availability Statement

The original contributions presented in the study are included in the article/supplementary material. Further inquiries can be directed to the corresponding author/s.

## Ethics Statement

The studies involving human participants were reviewed and approved by the Ethics Committee of Northwest Minzu University, Lanzhou, China. The patients/participants provided their written informed consent to participate in this study.

## Author Contributions

LF developed the study design, participated in and supervised data collection, performed the statistical analysis, and drafted the manuscript. RY conceived the study and revised the manuscript critically for important intellectual content. Both authors gave their final approval of the current version of the manuscript.

## Conflict of Interest

The authors declare that the research was conducted in the absence of any commercial or financial relationships that could be construed as a potential conflict of interest.
